# Supplementary material for: A Comprehensive Analysis of Chemical Composition and Anti-Inflammatory Effects of Cassava Leaf Extracts in Two Varieties in Manihot esculenta Crantz
Source: Int J Mol Sci. 2025 Apr 27;26(9):4140. doi: 10.3390/ijms26094140 (PMC12071432; doi:10.3390/ijms26094140)
Supplement: Supplementary file 1 [file ijms-26-04140-s001.zip › ijms-3486115-supplementary.pdf]

Supplementary Data:

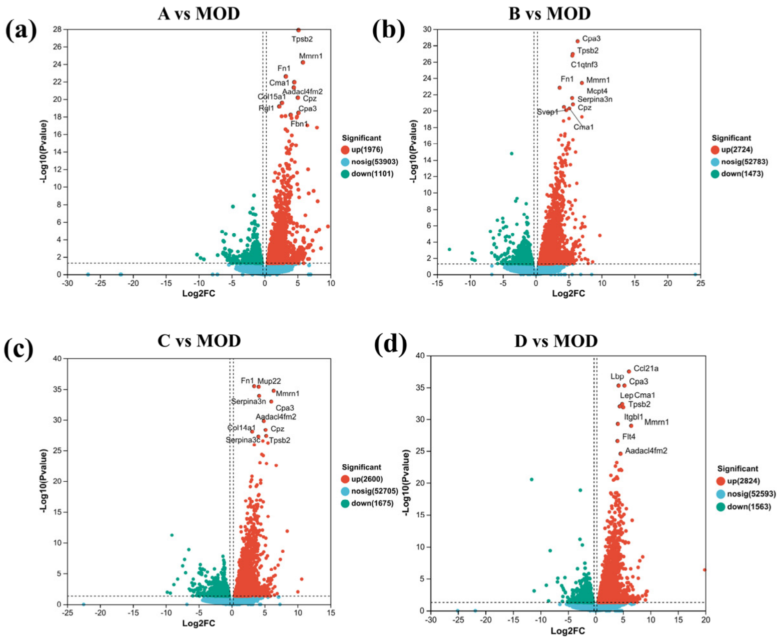

Supplementary Figure S1. Volcano maps of A, B, C and D groups.

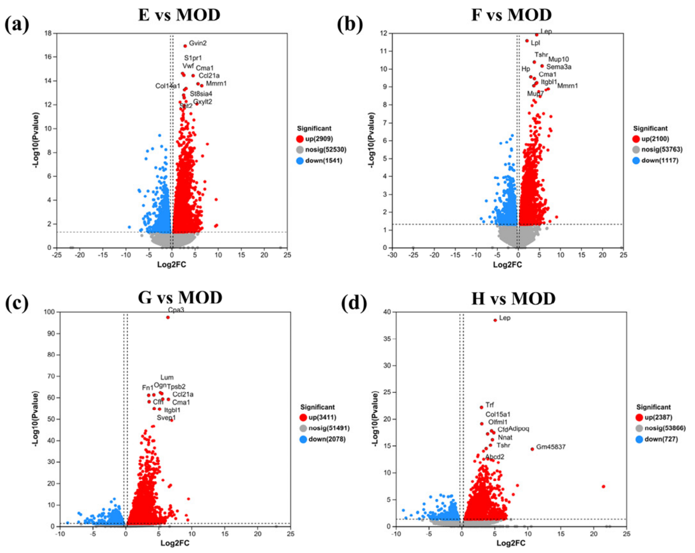

Supplementary Figure S2. Volcano maps of E, F, G and H groups.

Supplementary Table S1. The main components were obtained from G.L. leaves under different extraction conditions.

NO	RT [min]	Fragment ions (m/z) [M+H]$^+$	Fragment ions (m/z) [M-H]$^-$	Error (ppm)	Measured mass (m/z)	CAS	Formula	Identity	Relative content (%)			
									ethyl alcohol and then extracted with ethyl acetate	ethyl alcohol and then extracted with n-butanol	water and then extracted with ethyl acetate	water and then extracted with n-butanol
1	0.91	60.04508,70.0657,88.07607,106.08646		1.53	105.07914	111-42-2	$C_4H_{11}NO_2$	Diethanolamine	0.03	0.08	0.12	0.45
2	0.91	60.08143,87.04442,104.10716		3.02	103.06364	1956/12/2	$C_4H_9NO_2$	Y-Aminobutyric acid (GABA)	1.57	0.32	0.34	1.38
3	0.91	60.08144,104.10719		2.42	103.09996	62-49-7	$C_5H_{13}NO$	Choline	8.19	1.49	0.80	4.33
4	0.92	70.06564,88.07601,132.10175		-1.25	149.10501	104-74-3	$C_6H_{15}NO_3$	Triethanolamine	0.01	0.10	0.13	0.02
5	0.93		59.01375,75.00858,129.01917,195.0508	-1.24	196.05806	526-95-4	$C_6H_{12}O_7$	Gluconic acid	0.29	0.07	1.31	0.42
6	0.93		59.01377,71.01373,89.0243,179.05598	0.15	342.11626	612-11-1	$C_{12}H_{22}O_{11}$	α,α-Trehalose	2.01	0.30	0.04	0.25
7	0.94	56.05018,74.06056		0.62	119.05832	72-19-5	$C_4H_9NO_3$	L-Threonine	0.97	0.20	0.20	0.65
8	0.94		59.01375,71.01369,89.02427	-0.51	180.0633	6931-96-6	$C_6H_{12}O_6$	D-(+)-Mannose	16.32	5.17	2.09	7.47
9	0.95	60.08147,103.03922,116.07071,162.11226		-1.07	161.10502	540-73-8	$C_7H_{15}NO_3$	D-Carnitine	0.13	0.12	0.14	0.13

10	0.95		71.0137,89.02428,101.02422,181.07159	-0.78	182.0789	616-34-6	$C_6H_{14}O_6$	D-(-)-Mannitol	0.44	0.05	0.01	0.07
11	0.95	136.06158		-0.75	135.05439	65-21-7	$C_5H_5N_5$	Adenine	0.45	0.41	0.10	2.43
12	0.96	60.04503,88.03964		0.97	105.0427	56-40-6	$C_3H_7NO_3$	D-Serine	0.25	0.09	0.09	0.67
13	0.96		85.02934,111.00858,191.05595	-1.04	192.06319	77-97-6	$C_7H_{12}O_6$	D-(-)-Quinic acid	2.04	0.51	0.85	1.43
14	0.97	74.02415,87.05564		-0.32	132.05345	70-47-3	$C_4H_8N_2O_3$	Asparagine	0.06	0.01	-	0.16
15	0.97	84.04477,102.05513,130.04971		-1.19	147.05298	617-52-5	$C_5H_9NO_4$	L-Glutamic acid	1.71	0.46	0.31	1.40
16	0.98	94.06544,138.05475		-0.75	137.04758	612-14-3	$C_7H_7NO_2$	Trigonelline	8.12	2.12	1.37	7.03
17	0.99		73.02935,93.03441,111.04498,137.02425	-1.38	174.05258	138-59-0	$C_7H_{10}O_5$	Shikimic acid	0.15	0.03	0.02	0.08
18	0.99		71.01373,115.00352	-1.18	134.02137	617-52-5	$C_4H_6O_5$	DL-Malic acid	2.68	0.45	0.21	6.38
19	1	84.04478,130.04973		-0.95	146.069	56-86-0	$C_5H_{10}N_2O_3$	D-(-)-Glutamine	0.18	0.05	0.02	0.48
20	1	118.08625		1.05	117.0791	107-43-7	$C_5H_{11}NO_2$	Betaine	0.98	0.08	0.30	0.89
21	1.03	231.08345		13.7	230.07632	170-16-8	$C_{17}H_{10}O$	7-Oxobenz[de]anthracene	0.22	0.15	-	-
22	1.04		85.02934,129.03851	-0.97	130.02648	123-63-7	$C_5H_6O_4$	Glutaconic acid	0.53	0.15	0.18	0.92
23	1.06	61.0113,63.99836,79.02167		6.8	78.01447	67-68-5	C_2H_6OS	Dimethyl sulfoxide	0.01	0.06	0.13	0.07

24	1.06		85.02935,87.00860,111.00858	0.31	192.02706	77-92-9	$C_6H_8O_7$	Citric acid	1.85	1.82	4.27	5.48
25	1.07		89.02429	-0.99	90.0316	50-21-5	$C_3H_6O_3$	DL-Lactic Acid	0.49	0.29	0.59	0.35
26	1.08	84.04482,130.04979		-0.09	129.04258	142-04-1	$C_5H_7NO_3$	L-Pyroglutamic acid	0.68	0.32	0.17	0.50
27	1.1	124.03934		0.83	123.03213	59-67-3	$C_6H_5NO_2$	Nicotinic acid	0.09	0.01	0.04	0.08
28	1.21		73.02937,117.01923	-0.99	118.02649	110-15-6	$C_4H_6O_4$	Succinic acid	0.16	0.01	-	0.03
29	1.4	86.09681		0.12	131.09464	73-32-5	$C_6H_{13}NO_2$	L-Isoleucine	2.14	0.34	1.27	3.27
30	1.48		125.02419	-1.06	170.02134	149-91-7	$C_7H_6O_5$	Gallic acid	0.45	0.11	0.03	0.43
31	2.27	120.08074		-0.97	165.07882	63-24-6	$C_9H_{11}NO_2$	L-Phenylalanine	0.91	0.27	0.81	2.26
32	4.38	118.06512,146.05975		-1.15	187.06311	611-71-4	$C_{11}H_9NO_2$	Indole-3-acrylic acid	0.06	0.05	0.12	0.22
33	11.79	89.06008,133.08586,177.11174		-1.53	326.19357	68937-42-6	$C_{14}H_{30}O_8$	PEG n7	0.01	-	0.01	0.01
34	12.77	133.02827,137.05952,178.02576		-0.9	192.04209	532-41-9	$C_{10}H_8O_4$	Scopoletin	0.17	0.04	0.02	0.01
35	14.77	285.03888		-0.65	464.09517	482-35-5	$C_{21}H_{20}O_{12}$	Isoquercitrin	0.24	0.06	-	0.03
36	14.86	71.04966,85.02877,303.04916		-0.58	610.15303	153-18-4	$C_{27}H_{30}O_{16}$	Rutin	4.61	1.29	0.19	0.63
37	15.62	85.02879		-0.57	448.10031	482-32-7	$C_{21}H_{20}O_{11}$	Astragalin	0.20	0.05	-	0.04

38	15.63	71.04968,85.02879,287.05432		-0.62	594.1581	520-36-5	$C_{27}H_{30}O_{15}$	Kaempferol-3-O-rutinoside	3.01	0.76	0.16	
39	16.24	69.03405,139.11153		-0.78	138.10436	123-97-7	$C_9H_{14}O$	Isophorone	0.44	1.00	1.80	0.61
40	16.85	367.13544		-5.52	344.14655	170-58-9	$C_{17}H_{20}N_4O_4$	N--1,5-dimethyl-1H-pyrazole-3-carboxamide	-	0.16	0.01	0.04
41	17.17	69.07042,121.10114,139.1115,217.14299		-1.02	230.15157	111-26-2	$C_{12}H_{22}O_4$	Dimethyl sebacate	0.07	0.42	0.04	0.08
42	17.91	119.04916,192.13792		-1.22	191.13078	134-62-3	$C_{12}H_{17}NO$	DEET	0.02	0.05	0.11	0.06
43	18.1	149.02321,177.05441		-1.41	222.0889	119-32-2	$C_{12}H_{14}O_4$	Diethyl phthalate	-	0.08	0.10	0.09
44	18.24	309.13046		-1.98	286.17746	123-66-0	$C_{15}H_{26}O_5$	NP-011878	0.03	0.21	-	0.02
45	18.29	309.13022		-9.57	308.12304	123-67-1	$C_{16}H_{20}O_6$	NP-011199	0.06	0.08	0.21	0.11
46	18.59	57.07056,62.06063		-1.13	201.20904	111-76-2	$C_{12}H_{27}NO$	N, N-Dimethyldecylamine N-oxide	0.02	0.01	0.06	0.01
47	18.83	333.20383		-8.55	350.20633	123-68-2	$C_{20}H_{30}O_5$	6,8-dihydroxy-3-(10-hydroxyundecyl)-3,4-dihydro-1H-2-benzopyran-1-one	0.34	0.18	0.02	0.09
48	18.83	67.05481,81.0703,107.0857,275.2001		-1.65	292.20336	123-69-3	$C_{18}H_{28}O_3$	9S,13R-12-Oxophytodienoic acid	0.35	0.17	0.05	0.09
49	18.83		171.10251	0.22	328.22505	123-70-4	$C_{18}H_{32}O_5$	Corchorifatty acid F	0.60	0.29	0.05	0.16

50	19.03	441.18787		16.98	440.18043	123-71-5	$C_{19}H_{28}N_4O_6S$	N-[1-Amino-1-oxo-3-(2-thienyl)-2-propanyl]-1-(methoxyacetyl)-4-[(methoxyacetyl)amino]-2-piperidine carboxamide	0.02	0.34	0.03	0.04
51	19.04	105.07003		-1.8	386.17224	123-72-6	$C_{22}H_{26}O_6$	Bis (methylbenzylidene) sorbitol	0.32	0.83	0.35	0.23
52	19.36	353.2291		19.47	352.22193	123-73-7	$C_{22}H_{28}N_2O_2$	Methoxyacetyl fentanyl	0.11	0.06	0.03	0.02
53	19.37		139.11267,171.10251,211.13388,229.14439	0.29	330.24072	123-74-8	$C_{18}H_{34}O_5$	(15Z)-9,12,13-Trihydroxy-15-octadecenoic acid	0.19	0.13	0.01	0.04
54	19.52	55.05494,73.02892,83.08593		-1.51	226.12017	123-75-9	$C_{12}H_{18}O_4$	1,6-Hexanediol diacrylate	0.03	0.03	0.17	0.04
55	19.62	265.14035		-10.52	264.13338	123-76-0	$C_{15}H_{20}O_4$	NP-008274	0.06	0.11	0.01	0.04
56	19.74	119.08561,133.10114,217.1584,235.1689		-1.56	252.17215	123-77-1	$C_{15}H_{24}O_3$	NP-008095	0.01	-	0.04	0.02
57	19.76	119.08557		-1.72	392.30322	123-78-2	$C_{23}H_{40}N_2O_3$	AUDA	-	0.03	0.07	-
58	20.19	119.08555,145.10095,187.14772,233.15308		-1.58	232.14596	123-79-3	$C_{15}H_{20}O_2$	Atractylenolide II	0.02	0.02	0.05	0.04
59	20.38	57.07055,71.08606,85.10155,186.22125		-1.07	185.21415	123-80-4	$C_{12}H_{27}N$	Dodecylamine	0.22	1.33	3.25	1.38
60	20.56	70.04055		-1.13	307.14479	123-81-5	$C_{16}H_{22}ClN_3O$	Tebuconazole	0.01	0.01	0.07	0.05

61	20.67	318.17566		-9.32	332.19566	123-82-6	$C_{20}H_{28}O_4$	NP-002113	0.51	0.72	0.02	0.11
62	20.75		293.21228,311.22296	-0.97	312.22976	123-83-7	$C_{18}H_{32}O_4$	(±)9-HpODE	0.09	0.10	0.01	0.01
63	20.99	119.08559		-1.58	414.20358	123-84-8	$C_{24}H_{30}O_6$	Bis (4-ethylbenzylidene) sorbitol	0.11	0.15	0.30	0.14
64	21.09	216.8566		-8.9	334.21144	123-85-9	$C_{20}H_{30}O_4$	Bicyclo Prostaglandin E2	0.12	0.01	0.03	0.04
65	21.15	119.08555		-1.01	216.1512	123-86-0	$C_{15}H_{20}O$	(+)-ar-Turmerone	0.03	0.07	0.06	0.04
66	21.37	98.98435		-1.8	266.16422	123-87-1	$C_{12}H_{27}O_4P$	Tributyl phosphate	0.36	0.68	3.34	0.88
67	21.38	98.98446		-1.26	210.10183	123-88-2	$C_8H_{19}O_4P$	Dibutyl phosphate	0.04	0.09	0.29	0.08
68	21.46	149.02313		-1.75	278.15132	123-89-3	$C_{16}H_{22}O_4$	Diisobutylphthalate	0.36	1.44	9.29	1.87
69	21.5	347.21854		-8.48	346.21147	123-90-4	$C_{21}H_{30}O_4$	Methyl 1,4a-dimethyl-6-methylene-5-[2-(2-oxo-2,5-dihydro-3-furanyl) ethyl] decahydro-1-naphthalenecarboxylate	0.18	0.45	-	0.01
70	21.52	351.08948		-2.41	328.26057	123-91-5	$C_{19}H_{36}O_4$	1,4-dihydroxyheptadec-16-en-2-yl acetate	0.04	0.06	0.01	0.01
71	21.6	55.05495,79.0547,93.07021,275.20001		-1.5	292.20341	123-92-6	$C_{18}H_{28}O_3$	12-oxo Phytodienoic Acid	0.17	0.19	0.01	0.01
72	21.71		195.13884,275.20148	0.44	294.21962	123-93-7	$C_{18}H_{30}O_3$	13(S)-HOTrE	0.63	0.78	0.01	0.04

73	21.72	393.26022		-8.16	392.25307	123-94-8	$C_{23}H_{36}O_5$	NP-002336	0.01	0.15	-	0.01
74	22		223.20659,267.19641	-1.84	286.21388	123-95-9	$C_{16}H_{30}O_4$	Hexadecanedioic acid	0.04	0.33	0.65	0.16
75	22.08	317.20804		-9.59	316.20081	123-96-0	$C_{20}H_{28}O_3$	Cafestol	0.27	0.37	0.42	0.15
76	22.11	297.203		-1.63	274.21396	123-97-1	$C_{15}H_{30}O_4$	Monolaurin	0.02	0.10	0.04	0.01
77	22.18	301.21307		-9.32	318.21653	123-98-2	$C_{20}H_{30}O_3$	5-OxoETE	0.32	0.46	0.01	0.01
78	22.2	129.0181,139.00233,157.01286,185.08044		-1.57	402.22474	123-99-3	$C_{20}H_{34}O_8$	Citroflex A-4	-	0.01	0.67	0.06
79	22.27	422.19055		-7.04	448.27933	123-100-4	$C_{26}H_{40}O_6$	NP-006874	0.02	0.01	0.08	0.06
80	22.34		96.95989,265.14767	0.06	266.1552	123-101-5	$C_{12}H_{26}O_4S$	Dodecyl sulfate	-	-	0.11	0.04
81	22.62	303.22858		-8.13	320.23254	123-102-6	$C_{20}H_{32}O_3$	(+/-)11(12)-EET	0.27	0.31	0.36	0.17
82	22.74		205.15959	-0.07	250.15688	123-103-7	$C_{15}H_{22}O_3$	2-[(2S,4aR,8aS)-2-Hydroxy-4a-methyl-8-methylenedecahydro-2-naphthalenyl]acrylic acid	0.01	0.02	0.05	0.02
83	22.78	189.16348,203.17912,205.15837,409.34576		-1.19	472.3547	123-104-8	$C_{30}H_{48}O_4$	Corosolic acid	0.12	0.18	0.01	-

84	22.85	67.05479,81.07028,95.08577,109.10126		-2.09	292.23962	123-105-9	$C_{19}H_{32}O_2$	9(Z),11(E),13(E)-Octadecatrienoic Acid methyl ester	0.63	1.77	-	0.04
85	23.04	339.1774		-7.62	338.17036	123-106-0	$C_{18}H_{26}O_6$	NP-022470	0.06	0.03	0.21	0.41
86	23.05	57.07057,71.08607,95.08581,285.24179		-1.82	302.24516	123-107-1	$C_{17}H_{34}O_4$	2,3-dihydroxy propyl 12-methyltridecanoate	0.39	3.74	0.16	0.76
87	23.13	67.0548,81.0703,95.08579,109.10128		-1.89	306.2553	123-108-2	$C_{20}H_{34}O_2$	Linolenic acid ethyl ester	0.13	0.30	0.03	0.07
88	23.25	327.40707		-1.73	328.26079	123-109-3	$C_{19}H_{36}O_4$	2,4-dihydroxyheptadec-16-en-1-yl acetate	0.10	0.06	0.02	0.01
89	23.25	62.06068		-1.74	323.28187	123-110-4	$C_{20}H_{37}NO_2$	Linoleoyl Ethanolamide	0.05	0.01	0.28	0.02
90	23.31	351.24985		-8.84	350.24261	123-111-5	$C_{21}H_{34}O_4$	5-[5-(methoxycarbonyl)-5,8a-dimethyl-2-methylidene-decahydronaphthalen-1-yl]-3-methyl pentanoic acid	0.09	0.14	0.04	-
91	23.43		163.11272,339.23291	0.02	340.24024	123-112-6	$C_{23}H_{32}O_2$	2,2'-Methylenebis(4-methyl-6-tert-butyl phenol)	5.69	3.16	6.67	9.93
92	23.51	67.05479,81.07028,95.08576,263.23621		-1.88	354.27634	123-113-7	$C_{21}H_{38}O_4$	1-Linoleoyl glycerol	0.01	0.27	0.22	0.03
93	23.65	328.47882		16.19	426.29514	123-114-8	$C_{26}H_{38}N_2O_3$	N-(methyl)-2-methoxybenzamide	0.15	0.35	0.33	0.18

94	23.71		455.08777	-0.47	456.36013	123-115-9	$C_{30}H_{48}O_3$	Oleanolic acid	0.09	0.05	0.57	0.23
95	23.72	62.06067		-1.15	325.29771	123-116-0	$C_{20}H_{39}NO_2$	Oleoyl ethanolamide	0.04	0.06	0.33	0.04
96	23.79	55.05497,57.0706,69.07047,83.08598		-1.74	281.27137	123-117-1	$C_{18}H_{35}NO$	Oleamide	1.97	3.34	21.50	0.41
97	23.81	272.26706		-10.04	303.25317	67564-91-4	$C_{20}H_{33}NO$	Fenpropimorph	0.05	0.11	0.47	0.15
98	23.9		279.23282	-0.22	280.24017	60-33-3	$C_{18}H_{32}O_2$	Linoleic acid	0.13	0.09	0.01	0.01
99	23.95		96.95988,293.17914	0.64	294.18667	124-09-4	$C_{14}H_{30}O_4S$	Myristyl sulfate	0.11	-	0.01	0.10
100	23.96	69.07042,81.07029,95.08578,265.25189		-1.82	356.29201	591-05-9	$C_{21}H_{40}O_4$	Monoolein	0.45	0.98	1.28	0.11
101	24.11	109.10131		-1.59	424.36984	87-85-4	$C_{30}H_{48}O$	Lupenone	0.11	0.06	0.07	0.02
102	24.17	284.29404		-1.69	283.28704	124-22-2	$C_{18}H_{37}NO$	Stearamide	3.39	4.16	11.54	8.69
103	24.21	62.06068		-1.47	327.31325	6217-19-8	$C_{20}H_{41}NO_2$	Stearoyl ethanolamide	0.03	0.11	0.15	0.03
104	24.39		281.24854	-0.15	282.25584	638-90-6	$C_{18}H_{34}O_2$	Elaidic acid	0.06	0.08	0.03	0.01
105	24.4	413.26538		-7.99	412.25807	223460-87-5	$C_{26}H_{36}O_4$	5-[(10Z)-14-(3,5-dihydroxy phenyl) tetradec-10-en-1-yl] benzene-1,3-diol	0.12	0.30	0.30	0.05

106	24.4	149.02309		-1.99	390.27623	68515-49-1	$C_{24}H_{38}O_4$	Diisooctyl phthalate	0.21	0.04	0.19	0.31
107	24.42		171.25362	0.01	256.24023	1957/10/3	$C_{16}H_{32}O_2$	Palmitic acid	0.03	0.02	0.13	0.02
108	24.43	57.07063,71.08613,95.08589,341.3046		-1.75	358.30768	123-94-4	$C_{21}H_{42}O_4$	1-Stearoylglycerol	9.28	43.83	5.01	17.85
109	24.45	57.07056,58.02942,102.09155		-1.79	297.30263	67564-91-4	$C_{19}H_{39}NO$	Tridemorph	0.22	0.47	0.37	0.73
110	24.87	57.07055,70.06566,88.07603		0.05	284.27155	1957/11/4	$C_{18}H_{36}O_2$	Stearic acid	0.02	0.03	0.16	0.08
111	25.04	71.08607,85.10156,149.02312		-1.55	418.30766	119-07-3	$C_{26}H_{42}O_4$	Octyl decyl phthalate	0.09	0.09	0.19	0.02
112	25.13	69.07045,83.08596,97.10146,321.31445		-1.86	337.33384	112-84-5	$C_{22}H_{43}NO$	Erucamide	4.59	4.36	10.84	1.08
113	25.47	95.08588,109.10139,203.1792		-1.06	220.18248	139-08-2	$C_{15}H_{24}O$	(-)-Caryophyllene oxide	0.06	0.03	0.01	0.01
114	25.7	57.07057,88.07605,102.09156		-1.6	339.34957	112-84-5	$C_{22}H_{45}NO$	Docosanamide	0.22	0.12	0.01	0.21
115	27.17	81.07032,95.08581,107.08569,109.10131		-0.42	426.38599	87-85-4	$C_{30}H_{50}O$	Lupeol	0.36	0.31	0.03	-
		Total anti-inflammatory							38.27	14.73	27.19	19.54

Supplementary Table S2. The main components were obtained from P.L. leaves under different extraction conditions.

NO	RT [min]	Fragment ions (m/z) $[M+H]^+$	Mment ions (m/z) $[M-H]^-$	Error (ppm)	Measured mass (m/z)	CAS	Formula	Identity	Relative content (%)			
									ethyl alcohol and then extracted with ethyl acetate	ethyl alcohol and then extracted with n-butanol	water and then extracted with ethyl acetate	water and then extracted with n-butanol
1	0.908	60.0450, 70.0657, 88.0760, 106.0864		1.53	105.07914	105-59-9	$C_4H_{11}NO_2$	Diethanolamine	0.06	0.11	0.10	0.11
2	0.908	104.10716		3.02	103.06364	1956/12/2	$C_4H_9NO_2$	Y-Aminobutyric acid (GABA)	0.23	0.91	0.36	1.20
3	0.914	104.10717		2.42	103.09996	62-49-7	$C_5H_{13}NO$	Choline	0.85	2.05	1.68	5.58
4	0.92	150.11221		-1.25	149.10501	102-71-6	$C_6H_{15}NO_3$	Triethanolamine	0.14	0.06	0.18	-
5	0.933		195.0508,75.00858, 129.01917	-1.24	196.05806	526-95-4	$C_6H_{12}O_7$	Gluconic acid	0.05	0.18	0.69	1
6	0.934		89.02430, 59.01377, 71.01373, 179.05598	0.15	342.11626	612-11-1	$C_{12}H_{22}O_{11}$	α,α-Trehalose	0.21	0.44	0.12	0.83
7	0.939	74.06056		0.62	119.05832	72-19-5	$C_4H_9NO_3$	L-Threonine	0.12	0.27	0.26	0.38
8	0.94		59.01375, 71.01369, 89.02427	-0.51	180.0633	596-82-2	$C_6H_{12}O_6$	D-(+)-Mannose	5.51	11.38	4.3	12.73
9	0.948		181.07159, 71.01370, 101.02422, 89.02428	-0.78	182.0789	69-65-8	$C_6H_{14}O_6$	D-(-)-Mannitol	0.17	0.16	0.07	0.19

10	0.948	162.11226		-1.07	161.10502	541-15-7	$C_7H_{15}NO_3$	D-Carnitine	0.22	0.08	0.22	0.15
11	0.952	136.0615		-0.75	135.05439	60-61-7	$C_5H_5N_5$	Adenine	0.07	0.42	0.03	0.84
12	0.956	60.04503		0.97	105.0427	56-45-1	$C_3H_7NO_3$	D-Serine	0.04	0.22	0.41	0.54
13	0.961		191.0553	-1.04	192.06319	121-79-9	$C_7H_{12}O_6$	D-(-)-Quinic acid	0.44	0.83	0.62	1.89
14	0.972	84.04477		-1.19	147.05298	56-86-0	$C_5H_9NO_4$	L-Glutamic acid	0.52	1.41	1.31	4.06
15	0.976	138.05475		-0.75	137.04758	372-68-0	$C_7H_7NO_2$	Trigonelline	1.57	7.02	4.5	20.69
16	0.994	115.00352, 71.01373		-1.18	134.02137	617-48-1	$C_4H_6O_5$	DL-Malic acid	0.1	0.59	0.44	6.03
17	0.994		93.03441, 73.02935	-1.38	174.05258	138-59-0	$C_7H_{10}O_5$	Shikimic acid	0.19	0.51	0.14	0.48
18	0.998		84.04478, 130.04973	-0.95	146.069	56-85-9	$C_5H_{10}N_2O_3$	D-(-)-Glutamine	0.03	0.08	0.07	0.30
19	1.004	118.08625		1.05	117.0791	107-43-7	$C_5H_{11}NO_2$	Betaine	0.11	0.24	0.26	0.37
20	1.009		71.0137	-1.52	116.01078	110-17-8	$C_4H_4O_4$	Fumaric acid	-	0.03	0.02	0.25
21	1.033	231.0834		13.7	230.07632	218-28-5	$C_{17}H_{10}O$	7-Oxobenz[de]anthracene	0.30	0.94	-	-
22	1.04		85.0293	-0.97	130.02648	495-28-9	$C_5H_6O_4$	Glutaconic acid	0.14	0.54	0.53	2.74
23	1.055		191.0559	0.31	192.02706	77-92-9	$C_6H_8O_7$	Citric acid	1.75	4.32	3.55	6.78
24	1.056	79.0216		6.8	78.01447	67-68-5	C_2H_6OS	Dimethyl sulfoxide	0.06	0.34	0.01	0.16
25	1.074		89.0242	-0.99	90.0316	50-21-5	$C_3H_6O_3$	DL-Lactic Acid	0.41	0.65	1.11	0.55
26	1.079	84.0448		-0.09	129.04258	617-65-8	$C_5H_7NO_3$	L-Pyroglutamic acid	0.29	0.54	0.84	0.66
27	1.092	123.0553		0.92	122.04813	98-92-0	$C_6H_6N_2O$	Nicotinamide	0.01	0.06	0.69	0.02
28	1.101	124.0393		0.83	123.03213	59-67-6	$C_6H_5NO_2$	Nicotinic acid	0.03	0.02	0.05	0.09
29	1.21		73.0293	-0.99	118.02649	110-15-6	$C_4H_6O_4$	Succinic acid	0.02	0.07	0.01	0.08
30	1.268		71.0137	-2.37	72.02096	1979/10/7	$C_3H_4O_2$	Acrylic acid	-	0.04	0.02	0.07
31	1.404	86.0968		0.12	131.09464	73-32-5	$C_6H_{13}NO_2$	L-Isoleucine	0.05	0.19	0.48	0.75
32	1.483		125.0241	-1.06	170.02134	149-91-7	$C_7H_6O_5$	Gallic acid	0.08	0.26	0.32	0.81

33	2.27	120.0807		-0.97	165.07882	63-91-2	$C_9H_{11}NO_2$	L-Phenylalanine	0.02	0.23	0.29	0.49
34	4.375	146.0597		-1.15	187.06311	411-68-2	$C_{11}H_9NO_2$	Indole-3-acrylic acid	0.01	0.10	0.08	0.14
35	11.791	89.06		-1.53	326.19357	25322-68-3	$C_{14}H_{30}O_8$	PEG n7	0.01	0.01	0.03	0.01
36	12.769	133.0282		-0.9	192.04209	521-24-8	$C_{10}H_8O_4$	Scopoletin	0.04	0.27	0.01	0.23
37	12.929		353.0669	-0.71	594.15805	133-82-6	$C_{27}H_{30}O_{15}$	Vicenin II	-	0.05	-	0.07
38	13.379		300.9989	-1.9	302.00569	149-91-7	$C_{14}H_6O_8$	Ellagic acid	0.1	0.02	0.01	-
39	13.529		93.0343	-1.46	138.03149	69-72-7	$C_7H_6O_3$	Salicylic acid	0.04	0.01	0.04	0.04
40	13.888	319.0441		-0.77	480.09002	15648-86-9	$C_{21}H_{20}O_{13}$	myricetin 3-O-beta-D-galactopyranoside	0.05	0.37	0.01	0.15
41	14.765	61.029		-0.65	464.09517	482-35-9	$C_{21}H_{20}O_{12}$	Isoquercitrin	0.07	0.51	0.07	0.15
42	14.859	303.0491		-0.58	610.15303	153-18-4	$C_{27}H_{30}O_{16}$	Rutin	1.46	8.50	0.7	2.21
43	15.63	153.0179		-2.04	286.04716	520-18-3	$C_{15}H_{10}O_6$	Kaempferol	-	0.01	-	0.02
44	15.634	287.0543		-0.62	594.1581	17650-84-9	$C_{27}H_{30}O_{15}$	Kaempferol-3-O-rutinoside	0.53	2.64	0.26	1.17
45	15.784	317.0647		-0.79	624.16854	604-80-8	$C_{28}H_{32}O_{16}$	Isorhamnetin-3-O-rutinoside	0.06	0.28	0.04	0.25
46	16.237	139.11153, 69.0340		-0.78	138.10436	123-90-5	$C_9H_{14}O$	Isophorone	0.85	1.12	1.05	0.40
47	17.169	217.1431, 139.11159, 121.1012		-1.02	230.15157	111-12-6	$C_{12}H_{22}O_4$	Dimethyl sebacate	0.05	0.07	0.01	0.11
48	17.908	119.04916		-1.22	191.13078	134-62-3	$C_{12}H_{17}NO$	DEET	0.01	-	-	0.04
49	18.101	149.0232		-1.41	222.0889	84-66-2	$C_{12}H_{14}O_4$	Diethyl phthalate	0.01	0.11	0.02	0.04
50	18.394	102.0915, 158.1536		-0.87	157.14653	2564-86-2	$C_9H_{19}NO$	2,2,6,6-Tetramethyl-1-piperidinol (TEMPO)	0.30	-	0.06	-
51	19.036	105.07		-1.8	386.17224	69158-41-4	$C_{22}H_{26}O_6$	Bis (methylbenzylidene)	1.03	3.34	0.26	1.52

								sorbitol				
52	19.697		99.0813	-1.08	216.17231	301-16-7	$C_{12}H_{24}O_3$	12-Hydroxydodecanoic acid	0.02	-	0.06	-
53	19.757	119.08557		-1.72	392.30322	479413-70-2	$C_{23}H_{40}N_2O_3$	AUDA	0.01	0.02	0.04	0.01
54	20.112	119.08559		-1.77	414.20351	79072-96-1	$C_{24}H_{30}O_6$	Bis (4-ethylbenzylidene) sorbitol	0.15	0.08	0.53	0.03
55	20.187	119.08555, 145.1009, 233.1530		-1.58	232.14596	482-52-2	$C_{15}H_{20}O_2$	Atractylenolide II	0.04	0.03	0.05	0.03
56	20.376	186.2212, 71.0860		-1.07	185.21415	124-28-3	$C_{12}H_{27}N$	Dodecylamine	1.70	1.21	1.28	1.61
57	20.555	125.0152		-1.13	307.14479	652-49-1	$C_{16}H_{22}ClN_3O$	Tebuconazole	0.03	0.01	0.03	0.01
58	20.674	333.2028		-9.32	332.19566	-	$C_{20}H_{28}O_4$	NP-002113	0.04	0.29	0.03	0.09
59	20.852	71.086		-9.12	330.1801	84-61-7	$C_{20}H_{26}O_4$	Dicyclohexyl phthalate	0.17	0.62	0.62	0.34
60	21.375	98.9844		-1.26	210.10183	126-73-8	$C_8H_{19}O_4P$	Dibutyl phosphate	0.06	0.10	0.04	0.11
61	21.457	60.0450, 70.0657, 88.0760, 106.0864		-1.75	278.15132	84-68-0	$C_{16}H_{22}O_4$	Diisobutylphthalate	1.1	5.15	3.99	0.92
62	22.084	317.208		-9.59	316.20081	409-82-2	$C_{20}H_{28}O_3$	Cafestol	0.06	0.15	0.08	0.12
63	22.11	297.203		-1.63	274.21396	139-38-2	$C_{15}H_{30}O_4$	Monolaurin	0.09	0.03	0.11	0.01
64	22.155	284.3305		-1.68	283.32342	57-09-0	$C_{19}H_{41}N$	Cetrimonium	0.04	0.02	0.04	0.01

65	22.184	301.213		-9.32	318.21653	106154-18-1	$C_{20}H_{30}O_3$	5-OxoETE	0.04	0.19	0.01	0.02
66	22.195	129.0181		-1.57	402.22474	77-90-7	$C_{20}H_{34}O_8$	Citroflex A-4	-	0.12	0.06	0.03
67	22.34	363.2497		0.06	266.1552	151-21-3	$C_{12}H_{26}O_4S$	Dodecyl sulfate	0.03	0.01	0.94	0.09
68	22.718	309.2782		-1.69	325.29753	353-90-6	$C_{20}H_{39}NO_2$	Oleoyl ethanolamide	-	0.08	0.01	0.02
69	22.78	205.1583		-1.19	472.3547	76-83-5	$C_{30}H_{48}O_4$	Corosolic acid	-	0.05	-	-
70	22.847	67.0547, 81.0702, 95.0857		-2.09	292.23962	10219-70-2	$C_{19}H_{32}O_2$	9(Z),11(E),13(E)-Octadecatrienoic Acid methyl ester	0.62	1.62	0.01	0.2
71	23.048	57.0705		-1.82	302.24516	6284-43-1	$C_{17}H_{34}O_4$	2,3-dihydroxypropyl 12-methyltridecanoate	3.66	1.27	3.27	1.26
72	23.25	62.0606		-1.74	323.28187	2260670-52-6	$C_{20}H_{37}NO_2$	Linoleoyl Ethanolamide	0.17	0.14	0.14	0.02
73	23.254	327.407		-1.73	328.26079	24607-09-8	$C_{19}H_{36}O_4$	2,4-dihydroxyheptadec-16-en-1-yl acetate	0.01	0.03	0.05	0.01
74	23.505	67.0548, 81.0703, 95.0858		-1.88	354.27634	2258-92-6	$C_{21}H_{38}O_4$	1-Linoleoyl glycerol	0.27	0.46	0.1	0.01
75	23.705		455.0877	-0.47	456.36013	457-01-7	$C_{30}H_{48}O_3$	Oleanolic acid	0.21	0.30	0.06	0.26
76	23.792	69.0704, 57.0706, 55.0549		-1.74	281.27137	112-61-7	$C_{18}H_{35}NO$	Oleamide	0.15	0.07	0.02	0.23
77	23.813	272.267		-10.04	303.25317	298-81-7	$C_{20}H_{33}NO$	Fenpropimorph	0.12	0.95	0.27	0.08
78	23.902		279.2328	-0.22	280.24017	60-33-3	$C_{18}H_{32}O_2$	Linoleic acid	0.04	0.02	0.02	-
79	23.945		96.9598	0.64	294.18667	124-69-0	$C_{14}H_{30}O_4S$	Myristyl sulfate	0.23	0.19	0.30	0.15
80	23.963	69.0704,81.0702, 83.0859		-1.82	356.29201	592-28-7	$C_{21}H_{40}O_4$	Monoolein	1.35	0.92	1.02	0.10
81	24.025		183.01189	0.09	326.19159	139-09-8	$C_{18}H_{30}O_3S$	4-Dodecylbenzenesulfonic	1.09	0.16	3.15	0.43

								acid				
82	24.108	109.1012, 95.0857, 81.0702		-1.59	424.36984	470-98-3	$C_{30}H_{48}O$	Lupenone	0.08	1.17	0.12	0.03
83	24.176	274.2732		0.09	256.24025	1957/11/4	$C_{16}H_{32}O_2$	Palmitic acid	-	0.01	-	-
84	24.207	62.0606		-1.47	327.31325	111-57-9	$C_{20}H_{41}NO_2$	Stearoyl ethanolamide	-	-	0.13	0.04
85	24.298	95.0858, 109.1013, 107.0856		-0.66	426.38589	1985/1/8	$C_{30}H_{50}O$	Lupeol	0.02	0.66	0.02	0.05
86	24.39		281.2485	-0.15	282.25584	617-89-5	$C_{18}H_{34}O_2$	Elaidic acid	0.02	0.02	0.04	0.01
87	24.43	57.0706, 71.0861, 95.0858		-1.75	358.30768	123-94-4	$C_{21}H_{42}O_4$	1-Stearoylglycerol	68.67	29.91	55.59	15.64
88	25.04	149.0231, 71.0860		-1.55	418.30766	119-07-3	$C_{26}H_{42}O_4$	Octyl decyl phthalate	0.16	0.27	0.41	0.13
	Total anti-inflammatory								10.56	34.24	12.98	42.41

Supplementary Table S3. Common genes and regulation in A, B, C and D groups.

Gene ID	Gene	Gene description	*p*-value				Regulation situation
			A	B	C	D	
ENSMUSG00000058914	*C1qtnf3*	C1q and tumor necrosis factor related protein 3	1.23E-307	1.02E-307	2.44E-307	2.14E-09	up
ENSMUSG00000022225	*Cma1*	chymase 1, mast cell	8.16E-307	4.79E-306	2.88E-305	5.22E-08	up
ENSMUSG00000022371	*Col14a1*	collagen, type XIV, alpha 1	1.51E-306	6.06E-304	9.23E-305	3.91E-04	up
ENSMUSG00000028339	*Col15a1*	collagen, type XV, alpha 1	1.59E-305	9.54E-300	9.76E-304	1.49E-03	up
ENSMUSG00000001865	*Cpa3*	carboxypeptidase A3, mast cell	2.71E-302	1.43E-299	1.45E-303	2.54E-14	up
ENSMUSG00000036596	*Cpz*	carboxypeptidase Z	9.30E-302	3.14E-299	1.46E-303	1.66E-15	up
ENSMUSG00000027204	*Fbn1*	fibrillin 1	1.13E-301	2.37E-296	1.56E-301	1.01E-06	up
ENSMUSG00000026193	*Fn1*	fibronectin 1	3.48E-301	1.23E-295	4.13E-301	1.28E-02	up
ENSMUSG00000061068	*Mcpt4*	mast cell protease 4	1.82E-299	2.54E-292	4.20E-299	3.75E-18	up
ENSMUSG00000054641	*Mmrn1*	multimerin 1	9.89E-299	1.07E-291	1.63E-298	1.01E-29	up
ENSMUSG00000026482	*Rgl1*	ral guanine ucleotide dissociation stimulator, -like 1	1.46E-298	2.36E-291	2.06E-297	2.24E-15	up
ENSMUSG00000066361	*Serpina3c*	serine (or cysteine) peptidase inhibitor, clade A, member 3C	2.34E-298	7.44E-289	1.49E-296	3.20E-17	up
ENSMUSG00000021091	*Serpina3n*	serine (or cysteine)	2.45E-	8.20E-	2.18E-	5.66E-	up

		peptidase inhibitor, clade A, member 3N	297	289	296	24	
ENSMUSG00000028369	*Svep1*	sushi, von Willebrand factor type A, EGF and pentraxin domain containing 1	3.86E-297	1.50E-288	2.64E-296	1.03E-16	up
ENSMUSG00000033825	*Tpsb2*	tryptase beta 2	4.54E-297	1.58E-285	3.54E-296	1.34E-32	up

Supplementary Table S4. Common genes and regulation in E, F, G and H groups.

Gene ID	Gene	Gene description	*p*-value				Regulation situation
			E	F	G	H	
ENSMUSG00000039706	*Lep*	leptin	2.22E-10	9.76E-33	2.01E-32	4.04E-39	up
ENSMUSG00000042817	*Flt4*	FMS-like tyrosine kinase 4	4.12E-10	2.60E-27	1.60E-35	2.95E-13	up
ENSMUSG00000028339	*Col15a1*	collagen, type XV, alpha 1	1.08E-08	1.10E-11	1.74E-26	8.15E-20	up
ENSMUSG00000022878	*Adipoq*	adiponectin, C1Q and collagen domain containing	1.28E-08	4.57E-14	3.90E-47	1.84E-18	up
ENSMUSG00000022225	*Cma1*	chymase 1, mast cell	3.89E-15	4.21E-33	6.27E-60	9.92E-08	up
ENSMUSG00000061780	*Cfd*	complement factor D (adipsin)	1.35E-07	1.04E-14	9.54E-30	3.97E-18	up
ENSMUSG00000026365	*Cfh*	complement component factor h	1.04E-10	3.33E-21	9.54E-59	4.46E-07	up
ENSMUSG00000055782	*Abcd2*	ATP-binding cassette, sub-family	5.89E-07	2.14E-11	4.51E-31	3.47E-15	up

		D (ALD), member 2					
ENSMUSG00000094686	*Ccl21a*	chemokine (C-C motif) ligand 21A (serine)	1.87E-14	3.33E-38	7.90E-60	1.40E-06	up
ENSMUSG00000001865	*Cpa3*	carboxypeptidase A3, mast cell	8.90E-13	5.30E-36	3.94E-98	5.37E-06	up
ENSMUSG00000050368	*Hp*	haptoglobin	2.43E-05	2.45E-09	1.12E-15	1.51E-10	up
ENSMUSG00000041559	*Fn1*	fibronectin 1	8.98E-08	4.02E-16	1.13E-61	8.84E-05	up
ENSMUSG00000060594	*Lbp*	lipopolysaccharide binding protein	2.12E-09	5.34E-36	6.36E-32	1.44E-04	up
ENSMUSG00000022371	*Col14a1*	collagen, type XIV, alpha 1	4.64E-14	4.41E-13	5.70E-41	5.14E-04	up
ENSMUSG00000025534	*Gxylt2*	glucoside xylosyltransferase 2	1.72E-13	1.04E-09	3.47E-14	1.74E-03	up

Supplementary Table S5. The ABCDEFGH groups have both the GO term and the KEGG path.

Enrichment project	Common term or path
**GO**	Negative or positive regulation of mast cell activation<br>Attachment of a symbiont to a host<br>Collagen or elastic fiber tissue<br>Collagen metabolism (catabolism) process
**KEGG**	Complement and coagulation cascade<br>ECM-receptor interaction<br>PI3K-Akt signalling pathway<br>Complement and coagulation cascade
